# Neuronal surface P antigen (NSPA) modulates postsynaptic NMDAR stability through ubiquitination of tyrosine phosphatase PTPMEG

**DOI:** 10.1186/s12915-020-00877-2

**Published:** 2020-11-06

**Authors:** Sofía Espinoza, Sebastián B. Arredondo, Francisca Barake, Francisco Carvajal, Fernanda G. Guerrero, Fabian Segovia-Miranda, David M. Valenzuela, Ursula Wyneken, Alejandro Rojas-Fernández, Waldo Cerpa, Loreto Massardo, Lorena Varela-Nallar, Alfonso González

**Affiliations:** 1 Centro de Biología Celular y Biomedicina (CEBICEM), Facultad de Medicina y Ciencia, Universidad San Sebastián, 7510157 Santiago, Chile; 2 Centro de Envejecimiento y Regeneración (CARE), Facultad de Ciencias Biológicas, Pontificia Universidad Católica de Chile, 8330025 Santiago, Chile; 3 Institute of Biomedical Sciences (ICB), Faculty of Medicine and Faculty of Life Sciences, Universidad Andrés Bello, 8370146 Santiago, Chile; 4 Fundación Ciencia y Vida, 7780272 Santiago, Chile; 5 Laboratorio de Función y Patología Neuronal, Departamento de Biología Celular y Molecular, Facultad de Ciencias Biológicas, Pontificia Universidad Católica de Chile, 8330028 Santiago, Chile; 6 Centro de Excelencia en Biomedicina de Magallanes (CEBIMA), 6213029 Punta Arenas, Chile; 7 Regeneron Pharmaceuticals, Inc., Tarrytown, 10591 NY USA; 8 Laboratorio de Neurociencias, Facultad de Medicina, Universidad de los Andes, 7620001 Santiago, Chile; 9 Center for Interdisciplinary Studies of the Nervous System (CISNe), Universidad Austral de Chile, 5090000 Valdivia, Chile

**Keywords:** NMDA receptor, GluN2B Tyr1472, Tyrosine phosphatase PTPMEG/PTPN4, Synaptic plasticity, Memory, Postsynaptic densities, Ubiquitination, NSPA, *ZZEF1*, NPSLE

## Abstract

**Background:**

Cognitive dysfunction (CD) is common among patients with the autoimmune disease systemic lupus erythematosus (SLE). Anti-ribosomal P autoantibodies associate with this dysfunction and have neuropathogenic effects that are mediated by cross-reacting with neuronal surface P antigen (NSPA) protein. Elucidating the function of NSPA can then reveal CD pathogenic mechanisms and treatment opportunities. In the brain, NSPA somehow contributes to glutamatergic NMDA receptor (NMDAR) activity in synaptic plasticity and memory. Here we analyze the consequences of NSPA absence in KO mice considering its structural features shared with E3 ubiquitin ligases and the crucial role of ubiquitination in synaptic plasticity.

**Results:**

Electrophysiological studies revealed a decreased long-term potentiation in CA3-CA1 and medial perforant pathway-dentate gyrus (MPP-DG) hippocampal circuits, reflecting glutamatergic synaptic plasticity impairment in NSPA-KO mice. The hippocampal dentate gyrus of these mice showed a lower number of Arc-positive cells indicative of decreased synaptic activity and also showed proliferation defects of neural progenitors underlying less adult neurogenesis. All this translates into poor spatial and recognition memory when NSPA is absent. A cell-based assay demonstrated ubiquitination of NSPA as a property of RBR-type E3 ligases, while biochemical analysis of synaptic regions disclosed the tyrosine phosphatase PTPMEG as a potential substrate. Mice lacking NSPA have increased levels of PTPMEG due to its reduced ubiquitination and proteasomal degradation, which correlated with lower levels of GluN2A and GluN2B NMDAR subunits only at postsynaptic densities (PSDs), indicating selective trafficking of these proteins out of PSDs. As both GluN2A and GluN2B interact with PTPMEG, tyrosine (Tyr) dephosphorylation likely drives their endocytic removal from the PSD. Actually, immunoblot analysis showed reduced phosphorylation of the GluN2B endocytic signal Tyr1472 in NSPA-KO mice.

**Conclusions:**

NSPA contributes to hippocampal plasticity and memory processes ensuring appropriate levels of adult neurogenesis and PSD-located NMDAR. PTPMEG qualifies as NSPA ubiquitination substrate that regulates Tyr phosphorylation-dependent NMDAR stability at PSDs. The NSPA/PTPMEG pathway emerges as a new regulator of glutamatergic transmission and plasticity and may provide mechanistic clues and therapeutic opportunities for anti-P-mediated pathogenicity in SLE, a still unmet need.

## Background

Neuronal surface P antigen (NSPA) is a protein of unknown function originally discovered as a cross-reacting target of anti-ribosomal P protein autoantibodies (anti-P) that associates with neuropsychiatric manifestations, particularly psychosis and cognitive dysfunction (CD), in patients with systemic lupus erythematosus (SLE) [1]. CD is common among neuropsychiatric SLE (NPSLE) manifestations, affecting up to 80% of SLE patients [2]; it associates with a decreased quality of life [3] and has no targeted therapy at present [2]. Elucidating NSPA function in neurons can reveal mechanistic clues to better understand antibody-driven CD in NPSLE and could uncover new neuronal alterations leading to neuropsychiatric disease, from which more specific treatments might emerge.

Processes of learning and memory-encoding are possible thanks to synaptic plasticity that either strengthens or weakens synapses, as reflected in long-term potentiation (LTP) or long-term depression (LTD), respectively [4]. Mostly studied in the hippocampus, LTP and LTD are initiated by the activation of *N*-methyl-d-aspartate receptor (NMDAR) that results in long lasting increases or decreases in the function of α-amino-3-hydroxy-5-methyl-4-isoxazolepropionic acid receptor (AMPAR) [4]. AMPAR and NMDAR are ionotropic glutamatergic receptors highly enriched in the hippocampus and widely expressed in other areas of the brain participating in memory, emotion, and behavior [5, 6]. NMDAR receptors are conformed by two obligatory GluN1 subunits combined with two GluN2 (A, B, C, or D) or GluN3 (A and B) subunits, while AMPAR are composed of various combinations of four subunits (GluA1-GluA4) [7, 8]. The abundance of NMDAR and AMPAR at the postsynaptic density (PSD) crucial for LTP and LTD depends on their in-out diffusion and trafficking along endocytic routes, which are mainly controlled by phosphorylation modifications through yet little understood mechanisms [9, 10]. In different forms of synaptic plasticity, the role of glutamatergic receptors can be regulated by tyrosine phosphatases such as striatal-enriched protein tyrosine phosphatase (STEP), and the megakaryocyte protein tyrosine phosphatase (PTPMEG; also known as PTPN4) [11–15]. STEP has been shown to regulate Tyr dephosphorylation of NMDAR and AMPAR during LTP in the hippocampus [11, 15], while PTPMEG participates in synaptic plasticity associated with Tyr dephosphorylation of GluA2 during LTD in the cerebellum [13, 14]. Even though PTPMEG has been found to interact with GluN2 NMDAR subunits and to be present at the PSD [12], its role in the hippocampus remains unknown. Hippocampal memory processes also depend on neurogenesis involving proliferation, survival and differentiation of adult neural stem/progenitor cells (NPCs) [16]. Newly integrated neurons may participate in hippocampal functions that not only include learning and memory but also social behavior, anxiety, and stress regulation [17].

In NPSLE, anti-P antibodies originally described in patients with lupus psychosis have been found more recently to be associated with CD [1]. In vitro, anti-P antibodies induce calcium influx leading to apoptosis in neurons [18–20] and alter CA3-CA1 glutamatergic transmission and synaptic plasticity affecting the activity of AMPAR and NMDAR in hippocampal slices [20]. In vivo, anti-P antibodies injected into the cortex or hippocampus induce neuronal apoptosis very likely through glutamatergic excitotoxicity [18, 19]. However, a single injection of anti-P antibodies into the circulation accompanied by disruption of the blood brain barrier (BBB) impairs hippocampal-dependent spatial memory reflecting alterations in glutamatergic plasticity without enhancing apoptosis [19]. Therefore, anti-P antibodies may induce glutamatergic synaptic dysfunction accompanied or not by apoptosis likely depending on their concentration in the brain.

NSPA as target of anti-P antibodies and NMDAR as target of a subset of anti-dsDNA antibodies are currently considered essential mediators of antibody-driven CD in SLE patients [1, 2, 21–23]. So far, NSPA is the only recognized protein that mediates neuropathogenic effects of anti-P antibodies in NPSLE [1, 2, 20]. However, in contrast with the well-known function of ionotropic glutamatergic receptors and how they are affected as antibody targets [1, 21, 24, 25], the neuronal role of NSPA remains little understood. NSPA is a membrane protein that exposes a P-epitope to the cell surface and is expressed in neurons at regions involved in emotion, memory, and cognition [18]. NSPA is encoded by the unique *Zzef1* gene encompassing 2924 residues that include an anaphase promoter complex 10 (APC10) and two ZZ-type zinc finger domains [20]. These structural characteristics strongly point to NSPA as an E3 ubiquitin ligase [20]. The APC10 domain has only been described in E3 ubiquitin ligases [26], and even though the ZZ-type finger domains can be found in other proteins, it is also an important component of certain E3 ligases [27]. Protein ubiquitination catalyzed by E3 ligases is crucial in the regulation of AMPAR and NMDAR and consequently modulates glutamatergic synaptic transmission and plasticity [28]. Our previous studies approaching the function of NSPA characterized a knock-in mice that express a truncated NSPA (NSPA^tr/tr^, here called NSPA-TR) lacking the APC10 domain [20]. These NSPA-TR mice have alterations in glutamatergic plasticity reflected in impaired hippocampal LTP and poor performance in memory tests [20]. Interestingly, NSPA-TR mice have a decreased NMDAR-mediated transmission in the CA3-CA1 hippocampal circuit [20]. Therefore, NSPA seems to be required for NMDAR function and synaptic plasticity in memory [20]. NSPA function may hide unexpected aspects of glutamatergic synapse regulation and memory processes. It is thus important to first assess the effect of NSPA silencing, as unnoticed effects of truncated NSPA cannot be discarded. If the NSPA-TR phenotype is reproduced in NSPA knockout (NSPA-KO) mice, the unknown mechanism(s) linking NSPA to NMDAR function may be further explored. It is also interesting to define whether NSPA is required for other processes involved in memory, such as adult neurogenesis [29].

In this work, we used NSPA-KO mice and found that NSPA is not only involved in glutamatergic transmission and synaptic plasticity but also in adult neurogenesis. We also provide evidence pointing to NSPA as an E3 ubiquitin ligase and PTPMEG as one of its potential substrates. Furthermore, our results show that PTPMEG is degraded by the ubiquitin-proteasome system (UPS) impacting upon Tyr phosphorylation and PSD expression levels of NMDAR. Hippocampal PSDs of NSPA-KO mice have decreased levels of GluN2A and GluN2B, both NMDAR subunits that interact with PTPMEG. We propose that NSPA at the plasma membrane and downstream cytosolic PTPMEG constitutes a novel ubiquitin-based regulation system that contributes to synaptic plasticity and memory as determinants of NMDAR location at PSDs.

## Results

To understand the mechanism that involves NSPA with NMDAR function, we first performed experiments in mice lacking NSPA expression (NSPA-KO), aimed at discarding a previously unnoticed deleterious action of truncated NSPA on glutamatergic transmission. The results led us to also consider the role of NSPA in hippocampal adult neurogenesis, another process involved in memory [29], which is also sensitive to synaptic activity and plasticity [30]. Then, we used a heterologous system to test whether NSPA becomes ubiquitinated, as required for certain types of E3 ubiquitin ligases, and made biochemical analysis in the hippocampus to search for NSPA-regulated proteins. In particular, we focused on PTPMEG as a potential ubiquitination substrate that might regulate postsynaptic NMDAR levels through Tyr dephosphorylation.

### NSPA knockout (NSPA-KO) mice

Previous work showed that NSPA knock-in (NSPA-TR) mice expressing a truncated form of NSPA, which lacks the APC10 domain, perform poorly in the Morris water maze and memory flexibility tests and have depressed NMDAR-transmission and impaired LTP [20]. To discard that the truncated version of NSPA might act in a way unrelated to the native NSPA, we re-evaluated the NSPA phenotype in the hippocampus of knockout mice lacking any expression of NSPA.

NSPA full knockout mouse (Zzef1^tm2.1(KOMP)vlcg^) have 55 exons of the NSPA-encoding gene *Zzef1* replaced with a *LacZ* cassette, where β-gal staining reflects the activity of NSPA promoter. The β-gal staining pattern of NSPA-KO mice reproduced the pattern described in NSPA-TR mice [20]. In the hippocampus, NSPA is expressed in CA1, dentate gyrus, and ventral but not dorsal CA3 (Additional file 1: Fig. S1a). Reverse transcriptase-PCR using three primer pairs for different exons (Additional file 1: Fig. S1b) that detects NSPA mRNA in the hippocampus of wild type (NSPA-WT) showed absence of NSPA mRNA in the NSPA-KO mice (Additional file 1: Fig. S1c). Immunoblots with commercial anti-ZZEF1 and our anti-APC10 antibodies [20] could not detect NSPA protein in NSPA-KO mice (Additional file 1: Fig. S1c). These results corroborate the lack of NSPA expression in NSPA-KO mice.

### Impaired synaptic plasticity, neurogenesis and hippocampus-dependent memory in NSPA-KO mice

**Fig. 1:**
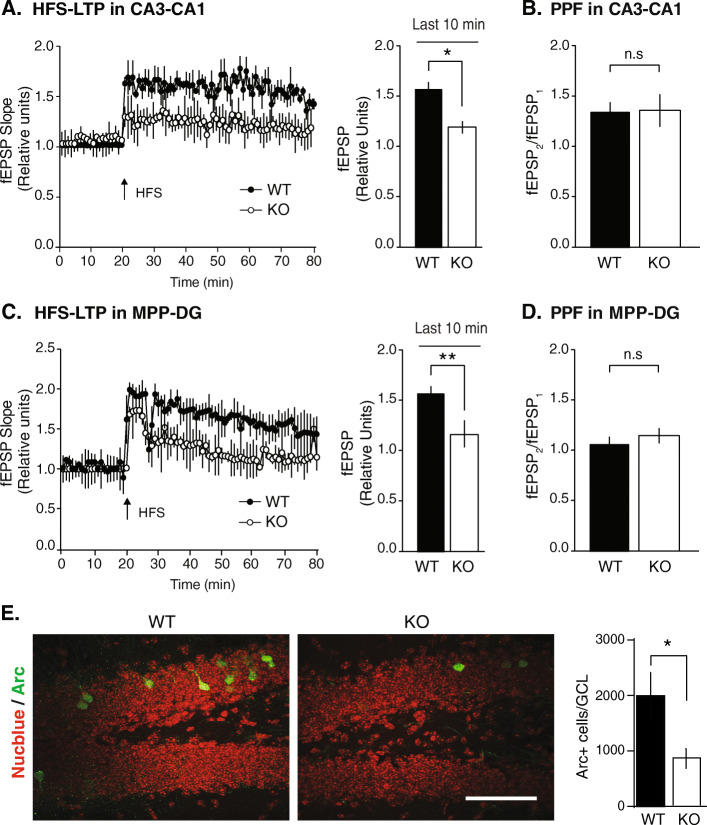
Impaired long-term potentiation (LTP) in CA1 and dentate gyrus (DG) regions of NSPA-KO mice. **a** LTP generated by high-frequency stimulation (HFS) in CA1 area shows a decreased fEPSP (quantified by slope and normalized by baseline) in NSPA-KO compared to WT mice. Graph corresponds to the last 10 min of recording (mean ± SEM; *n* = 9 slices; three animals per experimental group; *P < 0.05 by *t*-test.). **b** Paired pulse facilitation (PPF) of fEPSP shows no alterations in presynaptic activity in CA3-CA1 synapses of NSPA-KO mice (mean ± SEM; *n* = 7 slices; n.s, non-statistical differences, *t*-test). **c** Decreased LTP in MPP-DG of NSPA-KO compared with WT mice. Graph corresponds to the last 10 min of recording (mean ± SEM; *n* = 9 slices; three animals per experimental group; **P < 0.01 by *t*-test). **d** PPF indicates no alteration in presynaptic activity in MPP-DG synapses of NSPA-KO mice (mean ± SEM; *n* = 7 slices; n.s, non-statistical differences, *t*-test). **e** Immunofluorescence staining of Arc (Arc^+^) shows less neurons expressing this marker of neuronal activity in the DG of NSPA-KO mice (mean ± SEM; *n* = 3 mice per experimental group; *P < 0.05, *t*-test; Scale bar: 50 μm). Source data values are included in Additional file 2

NSPA-TR mice have impaired NMDAR-mediated neurotransmission without alterations in AMPAR-mediated transmission, nevertheless resulting in decreased synaptic plasticity of CA3-CA1 synapses [20]. Here, we expanded the LTP assays registering fEPSP not only in the Schaffer collateral-CA1 pathway (CA3-CA1 synapses), as before [20], but also in the medial perforant pathway synapses on dentate gyrus (DG) granule cells (MPP-DG synapses), which also express NSPA [20]. Similar to NSPA-TR mice [20], NSPA-KO mice showed decreased LTP in CA3-CA1 (Fig. 1a). Paired pulse facilitation assay discarded a presynaptic contribution (Fig. 1b). The MPP-DG synapses of NSPA-KO mice also showed decreased LTP (Fig. 1c) with unaltered presynaptic function (Fig. 1d). These results reinforce our previous proposal that NSPA plays a role in glutamatergic synaptic plasticity, here disclosed in two different hippocampal circuits related with memory [31].

To test whether NSPA is required for basal neuronal activity, we assessed the expression of Arc protein, an immediate-early gene product specifically targeted to regions of dendrites that receive direct synaptic stimulation [32]. Arc expression correlates with synaptic activity in glutamatergic neurons [33], increasing as a requirement for LTP and long-term memory consolidation [34, 35]. We found a decreased number of Arc-positive cells in the granule cell layer (GCL) of the NSPA-KO DG (Fig. 1e), compared with NSPA-WT mice. This indicates less DG neuronal activity in congruency with the decreased LTP observed in the MPP-DG synapses of NSPA-KO mice.

**Fig. 2:**
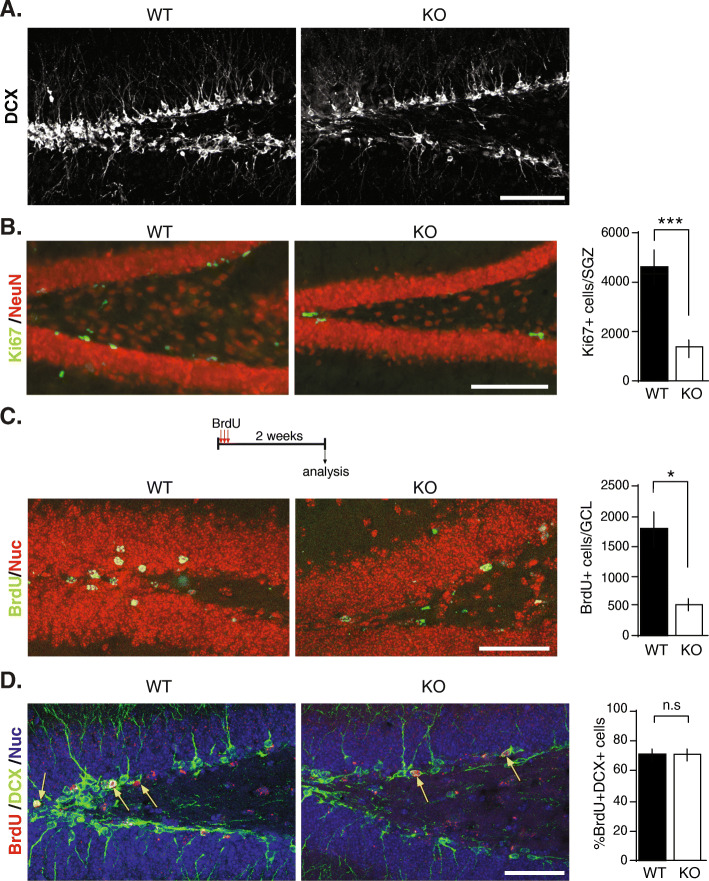
Decreased proliferation of neural progenitors in the dentate gyrus (DG). NSPA-KO mice. **a** Representative immunostaining of DCX in the DG of WT and NSPA-KO mice. Scale bar: 100 μm. **b** Representative immunostaining of Ki67 and the mature neuronal marker NeuN in the DG of WT and NSPA-KO mice. Scale bar: 100 μm. Quantification of total number of Ki67+ cells in the SGZ. (mean ± SEM; *n* = 10 mice per condition; ***P < 0.001, *t*-test). **c** Schematic representation of the experimental procedure. Animals received a daily dose of 100 mg kg^−1^ BrdU for 3 days and were sacrificed 2 weeks later. Immunostaining of BrdU in the DG of WT and NSPA-KO mice. Nuclei were stained with NucBlue (Nuc). Scale bar: 50 μm. Quantification of total number of BrdU+ cells in the granule cell layer (GCL) of the DG (mean ± SEM; *n* = 4 mice per condition; *P < 0.05, Mann-Whitney test). **d** Representative immunostaining of BrdU and DCX. Scale bar: 20 μm. Quantification of the percentage of BrdU-positive cells expressing DCX (mean ± SEM; *n* = 4 mice per condition; n.s, non-statistical differences, Mann-Whitney test). Source data values are included in Additional file 2

Adult hippocampal neurogenesis is regulated by DG neuronal activity and is involved in the hippocampal plasticity required for spatial learning, cognitive flexibility, object recognition and memory [29, 36, 37]. In the hippocampus, adult neurogenesis is restricted to the DG, where new neurons are generated from radial glia-like neural stem cells located within the subgranular zone (SGZ). After activation, these neural stem cells give rise to highly proliferative neural progenitor cells (NPCs), which then differentiate into immature neurons and subsequently to mature granule cells [16]. Induction of LTP in MPP-DG synapses promotes proliferation of NPCs and survival of adult-born neurons in an NMDAR-dependent manner [30, 38]. This process can be evaluated detecting doublecortin (DCX), which is expressed in newly generated neuroblasts and immature neurons [36]. We observed a decrease in DCX-positive cells in the DG of 2-month-old NSPA-KO compared with NSPA-WT mice (Fig. 2a), indicating a reduction in neurogenesis. To evaluate whether this reduction was due to a reduced proliferation, we assessed the mitotic marker Ki67. NSPA-KO mice showed a significant decrease in the total number of Ki67-positive cells in the SGZ compared to NSPA-WT mice (Fig. 2b). This indicates a reduced proliferation of hippocampal NPCs in the absence of NSPA. Then, to evaluate neuronal differentiation, mice received a daily dose of 100 mg/kg BrdU for 3 days and were euthanized 2 weeks later (Fig. 2c). We found a reduced total number of BrdU-positive cells in the granular cell layer (GCL) of the DG in NSPA-KO mice compared to that in NSPA-WT mice (Fig. 2c), without changes in the percentage of BrdU-positive cells expressing DCX (Fig. 2d). Altogether, these results indicate that NSPA-KO mice have decreased proliferation of NPCs, but unaffected differentiation of newborn cells into neurons.

**Fig. 3:**
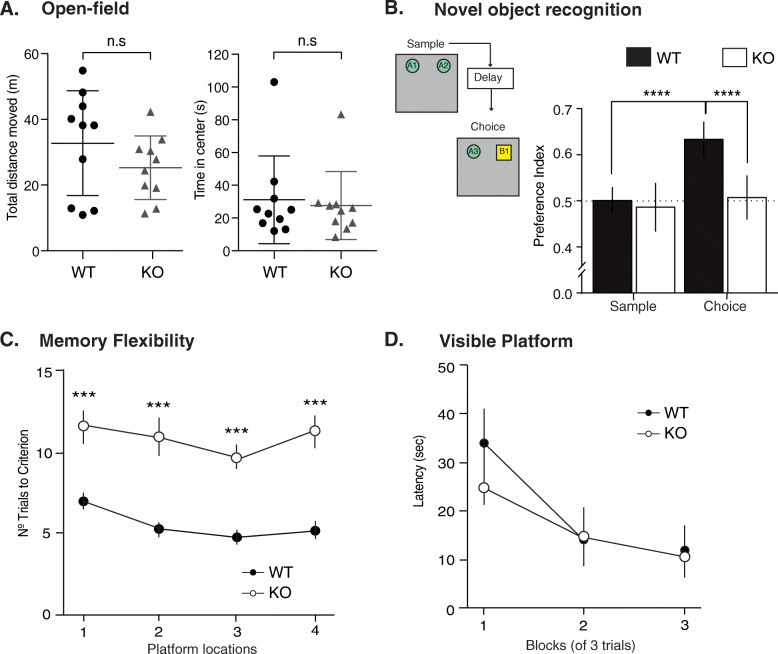
NSPA-KO mice have impaired hippocampal-memory-dependent tasks. **a** Open field test (novel environment) shows that WT and NSPA-KO mice have similar locomotor activity, as measured by the total distance moved, and also spent similar time in the center region (mean ± SEM; *n* = 10 mice per group; n.s, non-statistical differences, *t*-test). **b** Novel recognition (NOR) task comprising sample (10 min), delay (4 h), and choice (10 min) phases. In the scheme, A1 and A2 represent identical objects and B1 is a novel object. Preference index is the time spent with object divided by total exploration time. WT mice preferentially explored the novel object whereas NSPA-KO mice spent similar times in each object (mean ± SEM; *n* = 10 mice per group; ****P < 0.0001 versus WT mice by one-way ANOVA, followed by Bonferroni post hoc test). **c** Memory flexibility test shows a higher number of trials required by NSPA-KO compared with WT mice to meet criterion (mean ± SEM; *n* = 6 mice per group; ***P < 0.001 *t*-test). **d** Morris water maze test with visible platform indicates similar vision capability and general health of WT and NSPA-KO mice

Hippocampal LTP and adult neurogenesis are crucial in learning and memory [6, 29]. We tested locomotion, exploration, and anxiety reflecting general brain function and two behavioral tests that involve hippocampal memory function. The open field (OF) test that measures locomotion and anxiety-like behaviors [39] showed no alterations in NSPA-KO mice as measured by the total distance moved and similar amount of time spent in the center region of the OF arena compared with NSPA-WT mice (Fig. 3a). Hence, NSPA-KO mice have normal locomotion activity and no anxiety-like behavior. Then, we evaluated novel object recognition (NOR) memory [40] and found that NSPA-KO mice exhibit decreased preference for the novel object compared with NSPA-WT mice (Fig. 3b). Finally, a modified spatial memory paradigm task that evaluates episodic-like memory (memory flexibility) [41] showed poor performance of NSPA-KO mice (Fig. 3c), not due to vision capability problems (Fig. 3d). Therefore, NSPA-KO mice have impaired hippocampal-dependent memory, without anxiety-like behaviors or altered locomotion activity.

### NSPA as E3 ubiquitin ligase

**Fig. 4:**
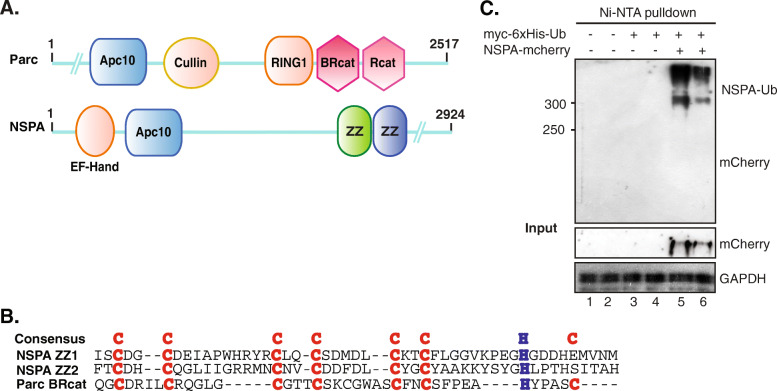
As characteristic of certain E3 ubiquitin ligases, NSPA becomes ubiquitinated in a cell-based ubiquitination assay. **a** Schematic representation of Cullin9/Parc and NSPA indicating linear domain organization. **b** NSPA ZZ-type finger domains (ZZ1 and ZZ2) sequence alignment with BRcat domain of Cullin9/Parc E3 ubiquitin ligase, conserved cysteine, and histidine residues are colored. **c** HEK293 cells transfected with NSPA-mCherry and myc-6xHis-ubiquitin and subjected to denaturing Ni-NTA pulldown followed by immunoblot with anti-mCherry antibodies clearly show NSPA ubiquitination. Lanes 1–2, 3–4, and 5–6 are duplicates of the same experiment

Ubiquitination is an important posttranslational modification involved in the regulation of glutamatergic transmission and synaptic plasticity [28]. NSPA has an APC10 domain exclusively found in E3 ubiquitin ligases and also contains two ZZ-type zinc finger domains that are included in some of these enzymes [20, 42] (Fig. 4a). NSPA ZZ-type domains have a cystein-rich pattern similar to the consensus pattern of the BRcat (also called IBR) domains found in RBR-type E3 ligases like Cullin9/Parc (Fig. 4b) and Parkin [42]. NSPA is then more closely related to RBR-type E3 ligases, which characteristically are first auto-ubiquitinated to then transfer the ubiquitin to their substrates [43]. We tested this possibility by assessing whether NSPA becomes ubiquitinated in a cell-based ubiquitination assay. HEK293 cells co-transfected with NSPA and myc-6xHis-Ub expression plasmids clearly showed ubiquitin-NSPA complexes, as detected by Ni-NTA affinity purification in strong denaturing conditions (Fig. 4c). Therefore, NSPA seems to be an E3 ubiquitin ligase with characteristics of RBR-type E3 enzymes [42]. Considering this posssibility, the altered synaptic plasticity of NSPA-KO mice might be due to abnormal levels of synaptic proteins that are directly degraded by the UPS or indirectly dependent on this system, as addressed below.

### Decreased levels of GluN2A and GluN2B NMDAR subunits in the hippocampal postsynaptic region of NSPA-KO mice

**Fig. 5:**
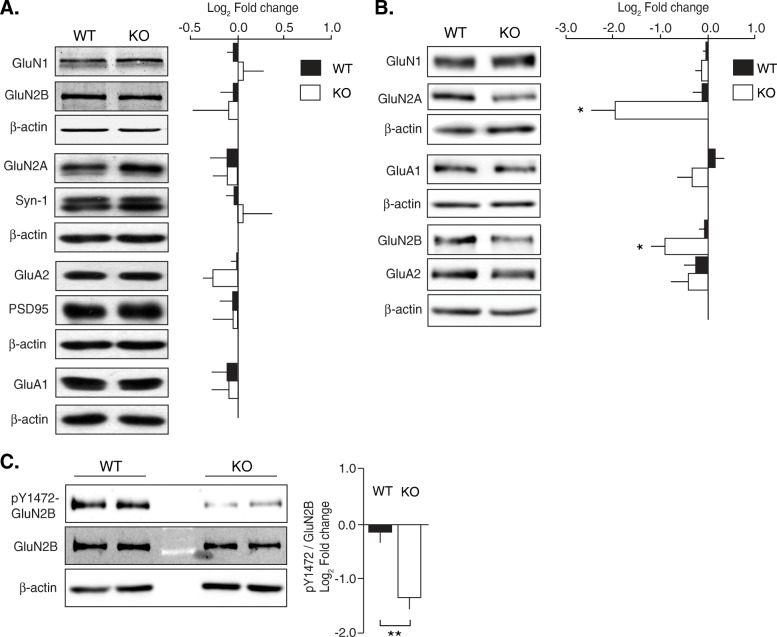
Decreased synaptic GluN2A and GluN2B expression accompanied with reduced GluN2B Y1472 phosphorylation in NSPA-KO hippocampus. **a** Total levels of synaptic proteins were analyzed by immunoblot in whole hippocampal lysates of WT and NSPA-KO mice; synaptic proteins levels were not different (mean ± SEM; *n* = 3 per group; n.s, non-statistical differences, *t*-test). **b** Hippocampal PSDs show lower levels of GluN2A and GluN2B in NSPA-KO compared with WT mice (mean ± SEM; *n* = 4 per group; *P < 0.05, **P < 0.01, *t*-test). **c** Immunoblot analysis and relative intensity of GluN2B Tyr1472P versus total GluN2B of hippocampal P2 fractions show lower levels of GluN2B Tyr1472P in NSPA-KO compared with WT mice (mean ± SEM; *n* = 8; **P < 0.01, *t*-test). Source data values are included in Additional file 2

Given the alterations in glutamatergic synaptic plasticity and memory in NSPA-KO mice and the possible role of NSPA as an E3 ubiquitin ligase, we evaluated the expression levels of hippocampal synaptic proteins. Immunoblot analyses of total hippocampal lysates showed no significant differences in synaptic receptors and scaffolding proteins between NSPA-WT and NSPA-KO mice (Fig. 5a). However, impaired LTP is most likely due to local alterations in synaptic proteins [6], and therefore, we isolated hippocampal synaptosomes and postsynaptic densities (PSDs) from both NSPA-WT and NSPA-KO mice. We verified the purity of isolated hippocampal PSDs by Western blot using the presynaptic marker VGlut1 and the postsynaptic marker PSD95 (Additional file 1: Fig. S2). Strikingly, we found a significant decrease of GluN2A and GluN2B subunits of NMDAR in NSPA-KO compared with NSPA-WT mice, both in synaptosomes (Additional file 1: Fig. S3) and PSDs (Fig. 5b). The lower levels of GluN2A and GluN2B subunits detected just in synaptosomes and PSDs of NSPA-KO mice, while maintaining similar levels in total hippocampal homogenates, reflect changes in the localization of these receptors rather than global expression modifications. This is also congruent with the decreased NMDAR-dependent neurotransmission previously detected in NSPA-TR mice [20]. Other relevant synaptic proteins such as PSD95, GluA1, and GluA2 AMPAR subunits remained relatively unchanged, indicating specificity of NSPA-mediated regulation. Ubiquitination of NMDAR subunits has been described and might be related with degradation pathways [28]. However, NSPA does not seem to be involved in NMDAR ubiquitination; otherwise, higher and not lower levels of NMDAR would have been found at the PSD. A defect in the ubiquitination of an NSPA substrate that selectively regulates the levels of GluN2A and GluN2B NMDAR subunits at the PSD might better explain these results.

### Decreased Tyr1472 phosphorylation of GluN2B subunit in NSPA-KO hippocampus

NMDAR postsynaptic abundance is critically regulated by the phosphorylation of the cytoplasmic tails of GluN2A and GluN2B subunits, including Tyr phosphorylated residues [44]. For instance, phosphorylation of GluN2B subunit at Tyr1472 (pTyr1472) prevents endocytosis and consequently enhances the stability and surface expression of NMDAR at the synaptic zone [9, 45]. An antibody specific to pTyr1472 [9, 45, 46] allowed us to assess the levels of GluN2B phosphorylated at this particular residue in hippocampal crude membrane fraction (P2), which we found decreased in NSPA-KO compared with NSPA-WT mice (Fig. 5c). Therefore, as reported in other conditions [9, 45, 46], dephosphorylation of GluN2B Tyr1472 may account for the decreased levels of NMDAR bearing this subunit at the PSD.

**Fig. 6:**
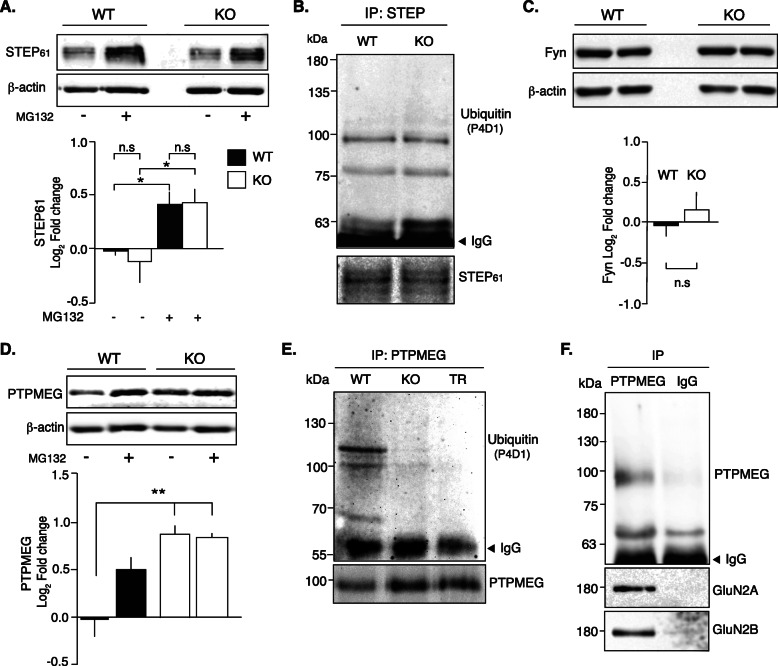
PTPMEG levels are increased due to less ubiquitination in hippocampal fractions of NSPA-KO mice. **a** STEP_61_ tyrosine phosphatase levels and **b** STEP_61_ ubiquitination analysis show no differences in hippocampal P2 fractions from WT and NSPA-KO mice. MG-132 treatment equally increased STEP_61_ levels in WT and NSPA-KO mice (mean ± SEM; *n* = 4; *P < 0.05; n.s, non-statistical differences, *t*-test). **c** Fyn kinase immunoblot show similar band intensities in WT and NSPA-KO mice (mean ± SEM; *n* = 3; n.s, non-statistical differences in *t*-test). **d** The levels of PTPMEG are increased in NSPA-KO compared with WT hippocampal P2 fractions prepared with DMSO (vehicle), which instead become similar under MG-132 treatment (mean ± SEM; *n* = 3; **P < 0.01, one-way ANOVA, followed Bonferroni’s post hoc test). **e** NSPA-dependent ubiquitination of PTPMEG. Immunoprecipitated PTPMEG from hippocampal P2 fractions followed by immunoblot with anti-PTPMEG or anti-ubiquitin (P4D1) show ubiquitinated PTPMEG only in WT and not in NSPA-KO or NSPA-TR mice (*n* = 3). **f** GluN2A and GluN2B subunits co-immunoprecipitated with PTPMEG, as shown by immunoprecipitation with anti-PTPMEG or control IgG from WT P2 fractions followed by immunoblot with anti-GluN2A and anti-GluN2B antibodies. Source data values are included in Additional file 2

These results suggest that enzymes that regulate GluN2B Tyr1472 phosphorylation have altered activities in NSPA-KO mice, likely due to ubiquitination-dependent defects affecting their levels. STEP_61_ is ubiquitinated by the E3 ligase Parkin [47], and its levels are UPS-controlled by degradation, impacting upon GluN2B Tyr1472 phosphorylation and NMDAR presence at the PSD [46, 48]. However, immunoblots of NSPA-WT and NSPA-KO synaptosomal P2 fractions showed no differences in STEP_61_ levels (Fig. 6a) and ubiquitination (Fig. 6b). We also evaluated tyrosine kinases of the Src family known to mediate tyrosine phosphorylation of GluN2 subunits, particularly Fyn that is the main subunit responsible for GluN2B Tyr1472 phosphorylation [49]. Neither Src (Additional file 1: Fig. S4) nor Fyn (Fig. 5c) showed level changes. Therefore, other enzymes distinct from STEP, Src and Fyn, would be involved in NSPA-dependent regulation of NMDAR Tyr phosphorylation.

### PTPMEG levels are increased due to impaired ubiquitination and UPS-mediated degradation in NSPA-KO hippocampus

PTPMEG is a Tyr phosphatase previously described to interact with GluN2 NMDAR subunits and to associate with the PSD, but its role in NMDAR Tyr phosphorylation remains unknown and even confusing [12]. We analyzed the levels of PTPMEG and also whether it is ubiquitinated and degraded by the UPS in an NSPA-dependent manner. Interestingly, hippocampal P2 fractions of NSPA-KO mice not only showed increased PTPMEG expression levels compared with NSPA-WT mice but also that proteasomal inhibition shielded this difference (Fig. 6d). Immunoprecipitation from crude hippocampal membrane fractions followed by immunoblot against ubiquitin revealed less ubiquitinated PTPMEG in NSPA-KO compared with NSPA-WT mice (Fig. 6e). The NSPA-TR mice expressing a truncated form of NSPA, which lacks the APC10 domain and have similar alterations as NSPA-KO mice [20], also showed reduced ubiquitinated PTPMEG (Fig. 6e). These results indicate that NSPA controls PTPMEG levels through ubiquitination and UPS-mediated degradation in the hippocampus. In this scenario, the absence of NSPA leads to increased PTPMEG levels very likely accounting for the decreased phosphorylation of GluN2B at Tyr1472.

### PTPMEG interacts with GluN2A and GluN2B subunits

Previous studies have described that PTPMEG interacts with the GluN2A and GluN2B subunits of NMDAR [12]. Accordingly, immunoprecipitation of PTPMEG from crude hippocampal P2 fraction of wild type mice followed by immunoblot against GluN2A and GluN2B subunits revealed the presence of both NMDAR subunits in the anti-PTPMEG immunoprecipitate (Fig. 6f). Therefore, PTPMEG interacts with GluN2A and GluN2B and most likely regulates Tyr phosphorylation of both subunits in the hippocampus. Tyr phosphorylation involving Tyr1472 in GluN2B and Tyr842 in GluN2A is known to regulate endocytosis and PSD abundance of both NMDAR subunits [49, 50]. Unfortunately, there is no available antibody against phosphorylated GluN2A Tyr842, but it seems very likely that PTPMEG interaction with GluN2A subunit has also a functional consequence in the phosphorylation status of this residue.

Taken together, all these results support a role of NSPA as an E3 ubiquitin ligase and PTPMEG as one of its substrates that regulates NMDAR levels at the PSD. PTPMEG expression levels are controlled by the UPS and are sensitive to NSPA expression, impacting upon memory processes in the hippocampus.

## Discussion

Since its discovery as a new neuronal surface protein [18], NSPA has been recognized as a mediator of the pathogenicity of anti-P autoantibodies that associate with psychosis and cognitive dysfunction in NPSLE [1, 2]. Our previous studies placed glutamatergic synaptic plasticity at the center of NSPA-mediated anti-P alterations leading to memory impairments [19, 20]. However, the mechanisms involving NSPA in synaptic function and memory processes remain little understood, limiting the possibility of targeted therapies against anti-P pathogenicity. The present study represents a significant step ahead. Our results obtained in NSPA-KO mouse and a heterologous cellular system point to NSPA as an E3 ubiquitin ligase involved in NMDAR expression at the PSD, which is indeed relevant in memory processes. PTPMEG qualifies as an NSPA-mediated ubiquitination substrate that is degraded by the UPS and very likely regulates NMDAR abundance at the PSD acting as a tyrosine phosphatase. We also found that NSPA is required for the process of adult neurogenesis, which depends on neuronal activity and can contribute to memory functions in the hippocampus [29, 36, 37]. These findings provide mechanistic basis for understanding the functional role of NSPA in neurons and as corollary suggest a pathway through which anti-P antibodies could drive neuronal dysfunctions and may be therapeutically targeted in NPSLE.

We first performed experiments similar to those previously reported in NSPA-TR mice using now a NSPA-KO (Zzef1^tm2.1(KOMP)vlcg^) mouse that completely lacks NSPA expression. NSPA-KO mice showed decreased synaptic plasticity in CA3-CA1 synapses and poor performance in memory flexibility. Therefore, our previous observations in NSPA-TR mice most likely resulted from defective NSPA function rather than a detrimental effect of the truncated protein. We extended the analysis and found that NSPA-KO mice also have an impaired LTP in MPP-DG synapses of dorsal hippocampus and performed poorly in object recognition tests, while open field assays did not detect anxiety-like behaviors. Object recognition tests not only reflect dysfunction in the hippocampus, as the memory flexibility test, but also in other brain regions such as the amygdala and cingulate cortex [51, 52], where NSPA is also expressed [18, 20]. All these results highlight a role of NSPA in glutamatergic synaptic plasticity associated with memory processes in the hippocampus and suggest other functional alterations remaining to be defined at different brain regions.

The impaired LTP in MPP-DG synapses of NSPA-KO mice led us to analyze neuronal activity at the DG and its related neurogenesis process. Neuronal activity promotes adult neurogenesis at the DG through secreted signaling factors that are sensed by neural progenitors [53, 54]. The expression of Arc in glutamatergic neurons is highly sensitive to synaptic activity [33] and is required for LTP and memory performance [34, 35]. We found a decreased expression of Arc indicating lower activity of neurons that lack NSPA at the DG. LTP at MPP-DG has been shown to promote proliferation of NPCs in an NMDAR-dependent manner without affecting neuronal differentiation [30, 38, 55]. Accordingly, NSPA-KO neural progenitors in the SGZ of DG showed reduced neurogenesis associated with proliferation but not differentiation defects. These results indicate a requirement of NSPA in the proliferation activity of neural progenitors. However, because LTP promotes survival of adult-born neurons [30], and glutamatergic signaling through NMDAR is crucial for the survival and integration of newborn neurons [55], it is possible that survival deficits of newborn neurons might also occur in the absence of NSPA. Indeed, an impaired hippocampal neurogenesis, as a form of cellular plasticity involved in learning and memory [29, 36] whose reduction associates with LTP impairments, and vice versa [38, 56], very likely contributes to plasticity and cognitive dysfunction in NSPA-KO.

Next, we focused on the potential role of NSPA in ubiquitin-mediated processes that control NMDAR function. E3 ubiquitin ligases are classified in RING, HECT, and RBR major classes [57]. NSPA has a characteristic APC10 domain of E3 ubiquitin ligases and two ZZ-type zinc finger domains that may represent RING domains similar to those in RBR-type E3 ligases [20, 42]. Although RBR-type E3 ligases usually have a catalytic unit conformed by three RING domains, RING1, in-between RING (BRcat or IBR), and RING2 (Rcat) [42, 57], some of these enzymes still promote ubiquitination when the RING1 domain is experimentally removed [58]. A biochemical hallmark of RBR-type E3 ligases is an active cysteine residue to which ubiquitin binds prior to its transfer to the substrate [42]. NSPA fulfill such criteria, as we demonstrated its ubiquitination in a cell-based assay. Further confirmation of NSPA as a member of the RBR E3 family would require defining whether conserved cysteines in ZZ-type domains are ubiquitinated. Nevertheless, structural and biochemical evidence, including the identification of an ubiquitination substrate, strongly suggest that NSPA might be the first transmembrane RBR-type E3 ubiquitin ligase.

NSPA function through ubiquitin-dependent pathways is an attractive possibility to explain why its absence leads to LTP impairment. Ubiquitination mediates protein degradation, signal transduction, and membrane trafficking associated with synapse maintenance, regulation, and organization in normal and altered brain functions [28]. Many proteins involved in synaptic plasticity are regulated by ubiquitination as specific substrates of particular E3 ubiquitin ligases [28]. The absence of NSPA as E3 ubiquitin ligase might result in higher levels of a substrate that is degraded by the UPS. We considered the cytosolic tyrosine phosphatase PTPMEG an interesting candidate. We found PTPMEG ubiquitinated in NSPA-WT but not in NSPA-KO mice, which accordingly showed increased PTPMEG levels in hippocampal fractions due to lower degradation by the UPS. Interaction between NSPA and PTPMEG is suggested by a wide-screen yeast two-hybrid analysis of brain-interacting proteins [59]. PTPMEG then qualifies as a substrate of NSPA-mediated ubiquitination, providing a link with NMDAR functions controlled by tyrosine phosphorylation.

NMDAR presence at the cell surface, including the PSD, is mainly regulated by a tyrosine phosphorylation system so far attributed to the tyrosine kinase Fyn and phosphatase STEP_61_ [9, 11, 45, 46, 49]. The most studied example is GluN2B Tyr1472 that conforms to an endocytic signal. Tyr1472 phosphorylation prevents endocytosis and thus stabilizes the NMDAR at the PSD, whereas its dephosphorylation leads to an endocytic decrease of NMDAR levels at the PSD [9, 45]. Strikingly, NSPA-KO mice showed not only decreased levels of GluN2B subunit but also lower phosphorylation of GluN2B Tyr1472 at the hippocampal PSD. Neither STEP_61_ nor Fyn showed evidence of changes congruent with a decreased GluN2B Tyr1472 phosphorylation. Instead, PTPMEG increased levels correlated well with the lower phosphorylated status of GluN2B Tyr1472. PTPMEG contains a PDZ (PSD95/Dlg/ZO-1) domain that interacts with proteins bearing a C-terminal PDZ-ligand sequence, including GluN2A and GluN2B [12, 14]. Our co-immunoprecipitation experiments corroborated that PTPMEG interacts with both GluN2A and GluN2B in the hippocampus. These assays cannot differentiate whether PTPMEG has a preference for either GluN2A or GluN2B subunits, as both can be found in the same tetrameric NMDAR [8]. Therefore, it seems very likely that PTPMEG dephosphorylates Tyr residues involved in endocytosis of both GluN2A and GluN2B subunits, with regulatory consequences upon the abundance of NMDAR at PSDs. Tyr phosphorylation status of GluN2A also regulates its cell surface stability [50], but there are no available antibodies against the endocytic-relevant phosphorylated Tyr in this subunit, as for GluN2B Tyr1472. However, we also found reduced GluN2A at the hippocampal PSD of NSPA-KO mice. All these data propose the following model. Increases in PTPMEG levels due to lower NSPA-mediated ubiquitination promote Tyr dephosphorylation and then removal of both GluN2B and GluN2A from the PSD, with consequences in hippocampal LTP that would normally be initiated by NMDAR activation [4].

The few studies on PTPMEG in neurons have shown a role in cerebellar motor learning and LTD synaptic plasticity [13, 14]. There are also two clinical cases of neurodevelopmental disorders seemingly involving hypofunctional PTPMEG [60, 61]. In one of these cases, a child with developmental delay, autistic features, and hypotonia was found to express a mutant PTPMEG that does not reach dendritic spines [61]. Our results are the first to evidence synaptic plasticity alterations associated with higher expression levels of PTPMEG in the hippocampus. The mechanism that regulates PTPMEG levels involves ubiquitination and degradation by the UPS, similar to other non-receptor tyrosine phosphatases [46, 62]. Interestingly, the unaffected PSD levels of PSD95 and AMPAR, whose degradation involves other ubiquitination systems [28], indicate a selectivity of the NSPA/PTPMEG pathway, so far including the NMDAR function.

Although we did not examine the NSPA/PTPMEG pathway directly in DG neural progenitors, NSPA and PTPMEG have been found to be expressed in DG [12, 20]. If DG neural progenitors do not express these proteins, they would still be under the influence of mature neurons [53]. Therefore, the NSPA/PTPMEG pathway involved in NMDAR function might be expected to influence DG adult neurogenesis. As our KO mice constitutively lack NSPA expression, we cannot discard indirect contributions of embryological disturbances to the dysfunctions described here. Conditional/inducible knockout mouse technology would be necessary to further define the role of NSPA and PTPMEG in neurogenesis and memory processes.

How might these results help to understand and manage the pathogenic role of anti-P autoantibodies in NPSLE patients? NSPA is the only known target that mediates neuropathogenicity of anti-P antibodies in lupus patients [20]. In the last three decades, clinical associations of anti-P antibodies with different manifestations of NPSLE have been reported [63]. The demonstration of an anti-P cross-reacting protein such as NSPA in the surface of neurons [18], together with anti-P neuronal effects in the brain [18–20, 64–66], indeed give strong support to a neuropathogenic role of anti-P in NPSLE [1, 2, 21]. Circulating anti-P antibodies may exert neuronal alterations in the brain after BBB disruptions [19, 66], which frequently occur in SLE [67]. The association of circulating anti-P with lupus psychosis [68] has been well documented [69, 70]. Clinical [23] and experimental evidence [19, 20] also involve anti-P in CD, which is the most frequent NPSLE manifestation [2], independent of depression [71], and may contribute to reduce health-related quality of life in SLE patients [3]. Both psychotic and cognitive disorders involve alterations in glutamatergic receptors [6, 24, 72] and can be elicited by a variety of different antibodies [21, 73]. Autoantibodies associated with autoimmune encephalitis can interfere with glutamatergic transmission directly targeting either NMDAR or AMPAR subunits, causing defective synaptic transmission and plasticity, memory dysfunction, and a diverse spectrum of neuropsychiatric manifestations [24]. The pathogenic mechanism of these antibodies often includes decreased location of NMDAR or AMPAR at the cell surface due to receptor internalization [24]. Similar anti-NMDAR antibodies that affect trafficking rather than channel activity have also been described in 20% of patients with psychosis due to schizophrenia [72]. In contrast, in patients with SLE, a subset of anti-double-stranded DNA antibodies that cross-react with NMDAR, originally described to bind GluN2A and GluN2B subunits in their open configuration [74], have more recently demonstrated preferential increase of synaptic transmission through GluN2A [25]. These lupus dsDNA/NMDAR antibodies also associate with spatial memory alterations and cognitive deficits in SLE patients [23, 75] and have the capability to alter spatial memory [76] and emotions [77] in mice, depending on where the BBB is breached. A long-term microglia-mediated damage also contributes to the pathogenesis of dsDNA/NMDAR antibodies [78]. In contrast with glutamatergic receptors as direct autoantibody targets, NSPA constitutes so far the only example of a target that mediates an antibody-driven indirect mechanism of NMDAR dysfunction. In mice, anti-P antibodies reproduce the phenotype of NSPA function absence leading to synaptic plasticity and memory impairments [19, 20], and also induce alterations of brain electrical activity likely eliciting glutamatergic dysfunctions [66]. Until now, empiric immunomodulatory therapy remains the main treatment for antibody-related neuropsychiatric disorders [2, 73]. However, improvements in understanding the pathogenic pathways of different antibodies are opening possibilities for evidence-based personalized and targeted therapies that are so far unavailable [73, 79]. For instance, recent evidence in animal models suggests increasing the availability of NMDAR at the cell surface through Ephrin-B2 receptor activation in certain anti-NMDAR autoimmune encephalitis [73], while selective inhibition of GluN2A subunits [25] and microglia [78] would be more adequate in SLE patients bearing dsDNA/NMDAR antibodies. Our present results guarantee further studies to disclose how anti-P antibodies might disturb the role of NSPA/PTPMEG/NMDAR pathway related with CD and psychiatric disorders in SLE. Interestingly, anti-P antibodies can elicit calcium influx in neurons [18, 20], and calcium-regulated calpain acutely activates PTPMEG through proteolytic cleavage [80]. These antibodies might then induce trafficking alterations leading to NMDAR internalization. PTPMEG as a functional link between NSPA and NMDAR function and the requirement of NSPA in adult neurogenesis provide processes upon which anti-P effects and targeted therapeutic alternatives in NPSLE can be tested.

## Conclusions

NSPA-dependent control of PTPMEG degradation involving ubiquitination provides a new pathway to be considered in processes of NMDAR-dependent synaptic plasticity and adult neurogenesis, as well as in the pathogenicity of anti-P autoantibodies in NPSLE. Previous studies have shown that autoantibodies directly targeting NMDAR and AMPAR are involved in neuropsychiatric manifestations of encephalitis syndromes [73], while a subset of anti-dsDNA antibodies cross-react with NMDAR and associate with CD in NPSLE [21, 22]. The NSPA/PTPMEG pathway constitutes an additional target for antibodies that can alter glutamatergic transmission through a different mechanism. NSPA can potentially contribute to ubiquitination-mediated synaptic transmission regulation, which is known to include processes of protein degradation, signal transduction, and membrane trafficking, underlying normal brain functions and brain disorders [28]. Future studies exploring these possibilities would shed new light on the mechanisms of synaptic plasticity and adult neurogenesis, their relationships with antibody-driven brain dysfunctions, and their potential use as suitable therapeutic targets that are much needed in NPSLE.

## Methods

### Antibodies

Mouse antibodies from UC Davis/NIH/NeuroMab Facility (UCLA, Davis, CA, USA) include anti-GluN1 (N308/48) (Cat#75-272, RRID:AB_11000180, 1:1000), anti-GluN2A (N327/95) (Cat#75-288, RRID:AB_2315842, 1:500), anti-GluN2B (N59/36) (Cat#75-101, RRID:AB_2232584, 1:1000), anti-GluA1 (N355/1) (Cat#75-327, RRID:AB_2315840, 1:1000), anti-GluA2 (L21/32) (Cat#75-002, RRID:AB_2232661, 1:1000), and anti-PSD95 (K28/43) (Cat#75-028, RRID:AB_2292909, 1:10,000), rabbit anti-phospho-GluN2B (Tyr1472) (Cell Signaling Technology Cat#4208, RRID:AB_1549657, 1:1000), rabbit anti-Synapsin I (Abcam Cat#ab8, RRID:AB_2200097, 1:10,000), mouse anti-Fyn (Santa Cruz Biotechnology Cat#sc-434, RRID:AB_627642, 1:1000), mouse anti-Src (17AT28) (Santa Cruz Biotechnology Cat#sc-130124, RRID:AB_2196197, 1:500), mouse anti-STEP (clone 23E5) (Millipore Cat#05-730, RRID:AB_11212456, 1:1000), rabbit anti-PTPMEG (Allele Biotechnology Cat# ABP-PAB-10818, 1:1000), mouse anti-ubiquitin (P4D1) (Santa Cruz Biotechnology Cat#sc-8017, RRID:AB_628423, 1:500), rabbit anti-ZZEF1 (Abcam Cat#ab176594,1:2000), rat anti-BrdU BU1/75 (ICR1) (Abcam Cat#ab6326, RRID:AB_305426, 1:250), rabbit anti-mCherry (Abcam Cat#ab167453, RRID:AB_2571870, 1:2500), rabbit anti-DCX (Cell Signaling Technology Cat#4604, RRID:AB_561007, 1:500), mouse anti-NeuN (Millipore, Cat.#MAB377, RRID:AB_2298772,1:500), rabbit anti-Arc (Abcam Cat#203056, RRID:AB_2827632, 1:100) and rabbit anti-Ki67 (Abcam Cat#ab15580, RRID:AB_443209, 1:500), mouse anti-GAPDH (Clone 6C5) (Millipore Cat#CB1001, RRID:AB_2107426, 1:1000), and mouse anti-β-actin (Abcam Cat#ab6276, RRID:AB_2223210, 1:10000). Secondary antibodies include horseradish peroxidase (HRP)-conjugated antibodies (Rockland) for immunoblot and Alexa (Molecular Probes) and DyLight (Abcam) conjugated antibodies for immunofluorescence. Affinity-purified rabbit anti-APC10 (12000) has been previously reported [20].

### LacZ knockout mice, genotyping and NSPA expression

C57BL/6NTac Zzef1^tm2.1(KOMP)vlcg^ (here called NSPA-KO) mice were engineered in Regeneron Pharmaceuticals Inc., New York, using Velocigene technology [81], replacing the entire coding region of the mouse *Zzef1* gene (128 kb) with ZEN-UB1 Cassette containing the LacZ gene that encodes β-galactosidase. Details are available at the Velocigene website (http://www.velocigene.com/komp/detail/10007). The following primers used in RT-PCR discarded NSPA mRNA expression: exons 4–5 (TATAGAAACGTCCTCCAACCC and GCTTCATCTTCAAACGTATCCA), exons 20–22 (GTCAACTGGTCATCTTCCTG and TCACACCTCTCATCAAATTCCA), exons 50–52 (TAGTGACTTTCAGCAGGACC and GATCTCAAACCCTGTCTGGA) for mice mRNA.

### Subcellular fractionation of mice hippocampi and immunoblotting

Hippocampi of 2- to 4-month-old WT or NSPA-KO mice were dissected on ice and homogenized in homogenization buffer (0.32 M sucrose, 0.5 mM EGTA, 5 mM Hepes, pH 7.4) supplemented with 4 μg/ml leupeptin, 4 mM PMSF, 4 μg/ml pepstatin, 25 mM NaF, and 100 mM Na_3_VO_4_ using a Potter homogenizer. In the experiments that inhibit the proteasome activity, 25 μM MG-132 or DMSO as control vehicle was additionally added. Homogenates were centrifuged twice at 1000×*g* for 10 min at 4 °C (H). The crude membrane fraction (P2) was obtained as described [82], centrifuging the supernatant (S1) at 12,000×*g* for 20 min at 4 °C. Synaptosomes were collected from the first sucrose step gradient at 1/1.2 M interphase and submitted to hypo-osmotic shock to release intracellular organelles. To obtain PSDs, synaptic membranes were collected from a second sucrose gradient at the 1/1.2 M interphase and delipidated in 320 mM sucrose, 1 mM dithiothreitol, 6 mM Tris-HCl (pH 8.1), and 0.5% Triton X-100. Protein concentrations were determined using the BCA assay (Pierce, Thermo Fisher Scientific). Immunoblots were made as described [18].

### Immunoprecipitation

P2 fractions from mice hippocampi solubilized in lysis buffer (50 mM Tris-HCl (pH 8.0), 150 mM NaCl, 2 mM EDTA, 1% Triton X-100) were subjected to immunoprecipitation with 1 μl of PTPMEG antibody or 10 μl of STEP antibody pre-bound to protein G-agarose; immunoprecipitated proteins were eluted with SDS sample buffer for subsequent immunoblotting.

### Behavioral tests

#### Open field and novel object recognition

All tasks were conducted in a behavioral suite. Trials were filmed and collected using a video tracking system coupled to Honestech TVR 2.5 program and analyzed offline in ANY-MAZE software (Stoelting Co, Wood Dale, IL, USA). Two- to 3-month-old male mice were used for behavioral tests. WT and NSPA-KO mice were placed in the center of a 40 × 40 × 40 cm box and allowed to explore for 10 min to assess their horizontal locomotion, with the center zone line at 10 cm from the edge (used for open field analyses) [39]. According to a reported test [40], there was another 10-min habituation to acclimatize the mice to the novel object recognition (NOR) field. On the next day, a 10-min familiarization phase (Sample) was conducted (two of object A). One of the objects was then replaced 4 h later (delay) with a novel one, and a testing period (choice) was conducted (one of object A and object B). Mice were allowed to explore freely during 10 min, object exploration was defined when a mouse directed its nose at an object within ∼ 2 cm or less and was actively investigating the object. For NOR, the “preference index” was calculated by the time spent to explore object A in the familiarization phase, or object B in the testing phase, compared to the total time explored in both objects.

#### Memory flexibility test

A modification of Morris water maze protocol [41] was used as described [20], in which learning criteria is to find a submerged platform with a new location each day in less than 60 s (escape latency) on 3 successive attempts, without overpassing 15 trials per day.

### BrdU administration and immunofluorescence

To analyze adult neurogenesis, 5-bromo-2′-deoxyuridine (BrdU, Sigma) was injected intraperitoneally (100 mg/kg) to 8 weeks mice for 3 days. Fourteen days after the last injection, mice were perfused with 4% paraformaldehyde and fixed brain sections were analyzed for BrdU and a neuronal marker immunofluorescence, as described [83]. BrdU-, Ki67-, or DCX-positive cells were counted using a fluorescence microscope (Olympus BX51, Tokyo, Japan) [83]. Double-labeled sections were analyzed by confocal laser microscopy (Leica SP8). Image analyses of maximal z-projections were made with ImageJ software (NIH, USA).

### Electrophysiology

#### Field excitatory postsynaptic potential (fEPSP)

Transverse slices (400 μm) from the dorsal hippocampus of 2- to 3-month-old male mice were prepared, maintained, and processed for electrophysiology adding picrotoxin (PTX; 10 μM) to suppress inhibitory GABA transmission [20]. Recordings were filtered at 2.0–3.0 kHz, sampled at 4.0 kHz using an A/D converter (National Instrument, Austin, TX, USA), and stored with the WinLTP program. To generate LTP in CA3-CA1 synapses, high-frequency stimulation (HFS) was used, consisting on 2 trains of 100 pulses at 100 Hz of stimuli, with an inter-train interval of 10 s [84]. LTP in MPP-DG synapses was induced by 4 trains of high-frequency stimulation at 100 Hz, 1 s in duration and 200 μs pulse width, with 5-min interval [85]. Data were collected and analyzed offline with pClamp 10 (Molecular Devices, San Jose, CA, USA).

#### Cell-based ubiquitination

HEK293 cells co-transfected with NSPA-mCherry and myc-6xHis-ubiquitin for 24 h were subjected to His-ubiquitin-based assay as described [86], with minor modifications. Briefly, cells were lysed in buffer A2 (6M guanidine chloride, 0.1 M Na2HPO4/NaH2PO4, and 10 mM immidazole, pH 8.0) and incubated with 100 μl of Ni-NTA agarose (QIAGEN, Valencia/CA, www.qiagen.com) for 3 h at room temperature. Ni-NTA beads were washed twice with buffer A2 and buffer A2/TI (1 volume of buffer A2 and 3 volumes of buffer TI) and once with buffer TI (25 mM Tris-HCl, 20 mM imidazole, pH 6.8). Proteins eluted with buffer ETI (50 mM Tris-HCl, 20 mM imidazole, pH 6.8) were analyzed by SDS-PAGE and immunoblotted with anti-mCherry antibodies.

### Statistical analysis

The software GraphPad PRISM Version 6.0c (San Diego, CA) was used for statistical analysis. Data are presented as mean ± SEM values and differences were analyzed with Student’s *t* test, Mann-Whitney, or one-way ANOVA followed by Bonferronni, as indicated. Statistical significances correspond to *P < 0.05, **P < 0.01, ***P < 0.001, and ****P < 0.0001.

## Supplementary information

**Additional File 1: Figure S1.** NSPA knockout mice (NSPA-KO). a Coronal slices of dorsal hippocampus from heterozygous NSPA-WT/KO mice stained for cresyl violet and β-Gal revealing activity of the NSPA promoter in CA1 and DG of the hippocampus. b Diagram of *Zzef1* gene showing the location of primers for RT-PCR spanning different exons (4-5, 20-22 and 50-52 exons) and the EF-Hand (amino acids 94-141), APC10 (251-380) and ZZ (1781-1828 and 1830-1877) domains. c Lack of NSPA expression in NSPA-KO mice demonstrated by RT-PCR in hippocampal mRNA extracts and immunoblot with anti-APC10 and anti-ZZEF1 antibodies in P2 synaptosomal fractions (Arrows indicates NSPA band). **Figure S2.** Purity of isolated hippocampal postsynaptic densities (PSDs). Presynaptic marker VGlut1 and postsynaptic marker PSD95 were used to verify the purity of PSDs by western blot. Crude hippocampal extract (H), supernatant obtained from hippocampal extract centrifugation (S2), crude membrane fraction (P2), synaptic membranes (SPM) and postsynaptic densities (PSD). **Figure S3.** Decreased levels of NMDAR GluN2A and GluN2B subunits in hippocampal synaptosomes of NSPA-KO mice. Hippocampal synaptosomes from WT and NSPA-KO mice were analyzed by immunoblot. Graph represents the intensity of the indicated proteins relative to beta-actin and shows significantly lower GluN2A and GluN2B levels in NSPA-KO compared with WT mice, while the levels of other proteins remain unaffected (mean ± SEM; *n* = 6 per group; *P < 0.05, **P < 0.01, *t*-test). **Figure S4.** Src kinase immunoblot show similar band intensities in WT and NSPA-KO mice (mean ± SEM; *n* = 4; n.s, non-statistical differences, *t*-test). Hippocampal P2 fractions from WT and NSPA-KO mice were analyzed by immunoblot. Source data values are included in Additional file 2.

**Additional file 2.** Excel sheet containing source data file for Fig. 1e, 2c, d, 5a, b, 6a, c, d and Additional file 1: Fig. S4.

## Data Availability

All data generated or analyzed during this study are included in this published article and its supplementary information files.

## Abbreviations

AMPAR: α-Amino-3-hydroxy-5-methyl-4-isoxazolepropionic acid receptor
BBB: Blood-brain barrier
DG: Dentate gyrus
DCX: Doublecortin
fEPSP: Field excitatory postsynaptic potentials
LTP: Long-term potentiation
MPP: Medial perforant pathway
NMDAR: *N*-methyl-d-aspartate receptor
NOR: Novel object recognition
NPSLE: Neuropsychiatric systemic lupus erythematosus
NSPA: Neuronal surface P antigen
OF: Open field
PSD: Postsynaptic density
PTPMEG: Megakaryocyte protein tyrosine phosphatase
SGZ: Subgranular zone
STEP: Striatal-enriched protein tyrosine phosphatase
SLE: Systemic lupus erythematosus
UPS: Ubiquitin-proteasome system
