# Supplementary material for: Neuronal surface P antigen (NSPA) modulates postsynaptic NMDAR stability through ubiquitination of tyrosine phosphatase PTPMEG
Source: BMC Biol. 2020 Nov 6;18:164. doi: 10.1186/s12915-020-00877-2 (PMC7648380; doi:10.1186/s12915-020-00877-2)
Supplement: Supplementary file 1 — Additional File 1: Figure S1. NSPA knockout mice (NSPA-KO). a Coronal slices of dorsal hippocampus from heterozygous NSPA-WT/KO mice stained for cresyl violet and β-Gal revealing activity of the NSPA promoter in CA1 and DG of the hippocampus. b Diagram of Zzef1 gene showing the location of primers for RT-PCR spanning different exons (4-5, 20-22 and 50-52 exons) and the EF-Hand (amino acids 94-141), APC10 (251-380) and ZZ (1781-1828 and 1830-1877) domains. c Lack of NSPA expression in NSPA-KO mice demonstrated by RT-PCR in hippocampal mRNA extracts and immunoblot with anti-APC10 and anti-ZZEF1 antibodies in P2 synaptosomal fractions (Arrows indicates NSPA band). Figure S2. Purity of isolated hippocampal postsynaptic densities (PSDs). Presynaptic marker VGlut1 and postsynaptic marker PSD95 were used to verify the purity of PSDs by western blot. Crude hippocampal extract (H), supernatant obtained from hippocampal extract centrifugation (S2), crude membrane fraction (P2), synaptic membranes (SPM) and postsynaptic densities (PSD). Figure S3. Decreased levels of NMDAR GluN2A and GluN2B subunits in hippocampal synaptosomes of NSPA-KO mice. Hippocampal synaptosomes from WT and NSPA-KO mice were analyzed by immunoblot. Graph represents the intensity of the indicated proteins relative to beta-actin and shows significantly lower GluN2A and GluN2B levels in NSPA-KO compared with WT mice, while the levels of other proteins remain unaffected (mean ± SEM; n = 6 per group; *P < 0.05, **P < 0.01, t-test). Figure S4. Src kinase immunoblot show similar band intensities in WT and NSPA-KO mice (mean ± SEM; n = 4; n.s, non-statistical differences, t-test). Hippocampal P2 fractions from WT and NSPA-KO mice were analyzed by immunoblot. Source data values are included in Additional file 2. [file 12915_2020_877_MOESM1_ESM.pdf]

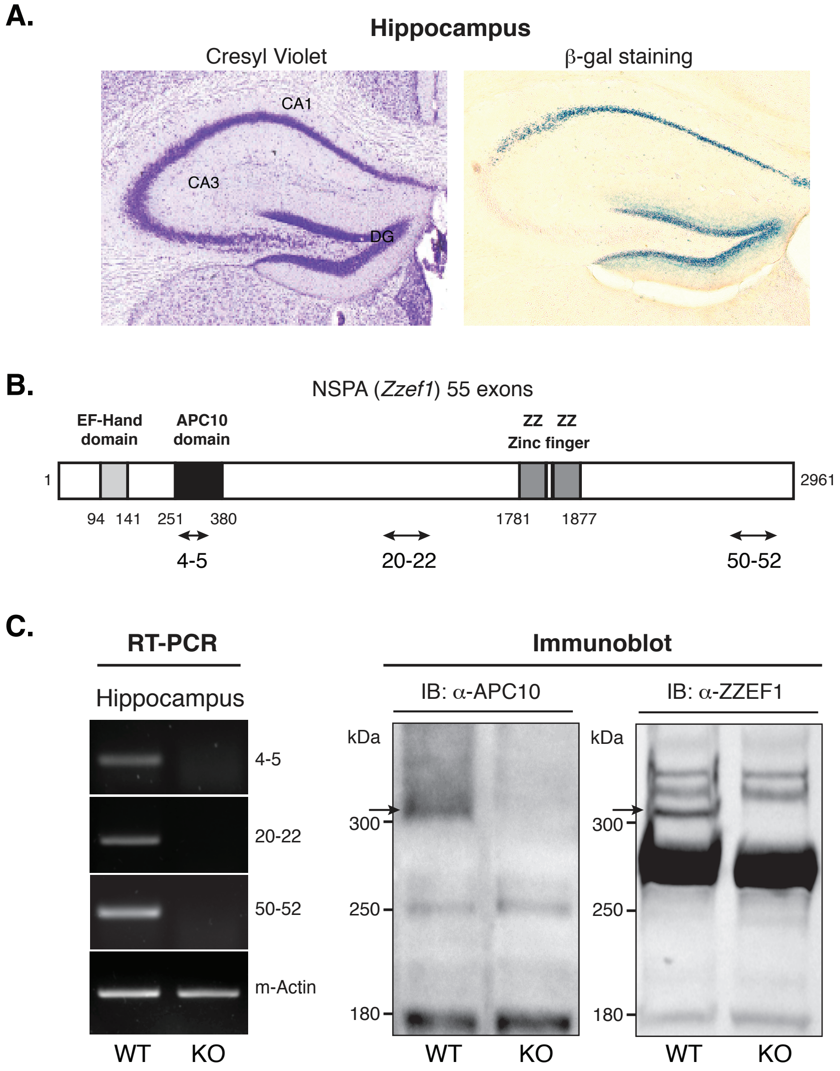

Additional file 1: Fig. S1. NSPA knockout mice (NSPA-KO). **a** Coronal slices of dorsal hippocampus from heterozygous NSPA-WT/KO mice stained for cresyl violet and β-Gal revealing activity of the NSPA promoter in CA1 and DG of the hippocampus. **b** Diagram of Zzef1 gene showing the location of primers for RT-PCR spanning different exons (4-5, 20-22 and 50-52 exons) and the EF-Hand (amino acids 94-141), APC10 (251-380) and ZZ (1781-1828 and 1830-1877) domains. **c** Lack of NSPA expression in NSPA-KO mice demonstrated by RT-PCR in hippocampal mRNA extracts and immunoblot with anti-APC10 and anti-ZZEF1 antibodies in P2 synaptosomal fractions (Arrows indicates NSPA band).

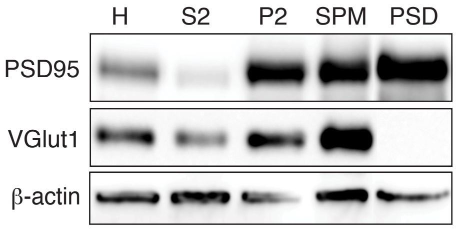

Additional file 1: Fig. S2. Purity of isolated hippocampal postsynaptic densities (PSD) from WT mice verified by western blot with presynaptic marker VGlut1 and postsynaptic marker PSD95. Crude hippocampal extract (H), supernatant obtained from hippocampal extract centrifugation (S2), crude membrane fraction (P2), synaptic membranes (SPM) and postsynaptic densities (PSD).

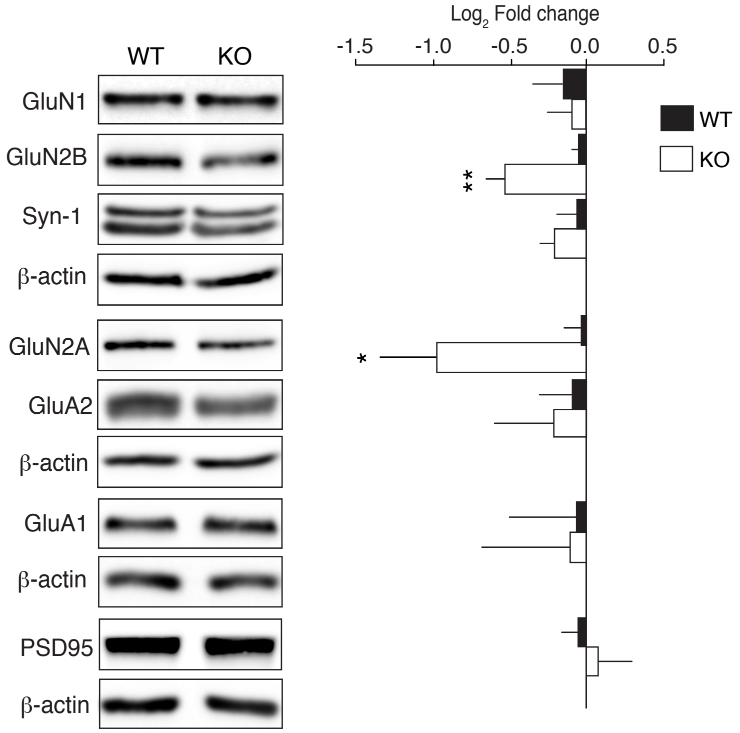

Additional file 1: Fig. S3. Decreased levels of NMDAR GluN2A and GluN2B subunits in hippocampal synaptosomes of NSPA-KO mice. Hippocampal synaptosomes from WT and NSPA-KO mice were analyzed by immunoblot. Graph represents the intensity of the indicated proteins relative to beta-actin and shows a significantly lower GluN2A and GluN2B levels in NSPA-KO compared with WT mice, while the levels of other proteins remain unaffected (mean ± SEM; $n = 6$ per group; $^*P < 0.05$, $^{**}P < 0.01$, t-test).

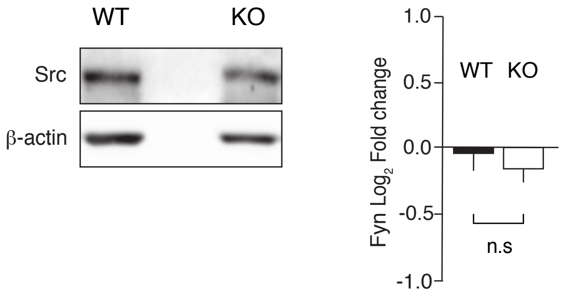

Additional file 1: Fig. S4. Src kinase immunoblot show similar band intensities in WT and NSPA-KO mice (mean ± SEM; *n* = 4; n.s, non-statistical differences, *t*-test). Hippocampal P2 fractions from WT and NSPA-KO mice were analyzed by immunoblot.
